# Resilience in caregivers of people with mild-to-moderate dementia: findings from the IDEAL cohort

**DOI:** 10.1186/s12877-023-04549-y

**Published:** 2023-12-05

**Authors:** Anthony Martyr, Jennifer M. Rusted, Catherine Quinn, Laura D. Gamble, Rachel Collins, Robin G. Morris, Linda Clare

**Affiliations:** 1 Centre for Research in Ageing and Cognitive Health, Faculty of Health and Life Sciences, University of Exeter, St Luke’s Campus, Exeter, UK; 2 School of Psychology, University of Sussex, Brighton, UK; 3 Centre for Applied Dementia Studies, University of Bradford, Bradford, UK; 4 Wolfson Centre for Applied Health Research, Bradford, UK; 5 Population Health Sciences Institute, Newcastle University, Newcastle upon Tyne, UK; 6 Department of Psychology, King’s College London Institute of Psychiatry, Psychology and Neuroscience, London, UK; 7 NIHR Applied Research Collaboration South-West Peninsula, Exeter, UK

**Keywords:** Carers, Carer stress, Competence, Personality, Resilient

## Abstract

**Objectives:**

A novel model of resilience was tested in caregivers of people with mild-to-moderate dementia and was extended to explore whether including self-efficacy, optimism, and self-esteem improved its predictive value.

**Design:**

Cross-sectional.

**Setting:**

Data from the IDEAL cohort were used.

**Participants:**

The study comprised 1222 caregivers of people with dementia.

**Measurements:**

A composite resilience score was calculated from five measures. Multivariable regressions were used to investigate factors associated with resilience.

**Results:**

Greater resilience was associated with being older, being male, and caregiving for older people with dementia. Greater resilience was also observed when people with dementia had fewer functional difficulties and/or fewer neuropsychiatric symptoms, there was a stronger dyadic relationship, and the caregiver had fewer social restrictions, less neuroticism, and greater perceived competence. Surprisingly, caregiver self-efficacy, optimism, and self-esteem were unrelated to resilience.

**Conclusion:**

Caregivers of people with mild-to-moderate dementia generally scored well for resilience. Resilience was associated with both the personal characteristics of caregivers and level of care need among people with dementia. Future work is needed to determine whether the caregivers in this cohort appeared resilient because the care recipients had relatively low care needs and consequently placed fewer demands on caregiver well-being than would be the case where dementia is more advanced.

**Supplementary Information:**

The online version contains supplementary material available at 10.1186/s12877-023-04549-y.

## Background

Globally, there are over 2.5 billion informal caregivers (subsequently referred to as caregivers), with approximately 13.6 million living in Britain [1] and 48 million living in the United States of America [2]. In Britain caregivers of people with mild-to-moderate dementia spend an average of 36 h per week caregiving [3] and the number of hours spent caregiving increases over time [4]. This type of care typically includes the same tasks as those undertaken by formal paid caregivers, such as personal care (washing, dressing, etc.) and practical care (shopping, laundry, supervision, etc.). Caregivers contribute towards healthcare expenditure savings of £193 billion per year [5], reducing hospitalizations, preventing residential care placements, and improving health outcomes, well-being, and quality of life for people with dementia [6–8], but this is often at the expense of their own physical and mental health [9].

A dementia diagnosis, with the prospective loss of independence and functional abilities for people with dementia [10, 11], presents a major challenge to psychosocial resources both for people with dementia and their caregivers [12–16]. There is growing interest in the concept of resilience and how this can impact both the quality of care provided and caregiver well-being [17, 18]. Resilience has no fixed definition, and has been variously operationalized as the capacity to adapt positively- while dealing with loss, burden, hardship, or adversity- to changing life circumstances in a manner that protects psychological and physical health [19, 20]. Resilience in caregivers of people with dementia has been described as reflecting how well a caregiver manages the level of subjective burden and the frequency and severity of the care needs of the person with dementia [21]; i.e., a caregiver who is managing well but who has high levels of burden and is supporting a person with dementia who has high care needs would have high resilience whereas a caregiver with a similar set of characteristics and situation who is not managing well has low resilience. An individual’s level of resilience therefore derives from the resources available to enable an adaptive response to challenging circumstances, including social and interpersonal relationships, and personal attributes [17]. Qualitative studies have explored factors associated with resilience in caregivers of people with dementia and these highlight the multiplicity of protective factors and adaptive processes that contribute to resilience. Ability to derive meaning from caregiving, sense of coherence and identity, and positive emotions in caregiving associate with greater resilience [22]. This is consistent with the outcomes from a systematic review of resilience in family caregivers of people with dementia [23]. Despite significant differences in the way resilience has been measured across the different studies, the review concluded that resilience is multifaceted and was influenced by numerous interrelated factors that broadly comprised three areas: sociocultural factors (age, education, ethnicity, sex, religiosity, socioeconomic position), context (kinship ties, level and time spent caregiving, respite opportunities, social network, social and professional support), and psychological dimensions (coping style, finding meaning in caregiving, personality) [23]. This multifactorial formulation is reinforced in other reviews [18, 19].

Earlier studies have typically investigated resilience using a single measure [24] which is problematic as resilience is multifaceted and multidimensional [20]. To mitigate this the present study calculated a composite resilience index from five contributory measures utilizing data from the Improving the experience of Dementia and Enhancing Active Life (IDEAL) cohort [15]. The first aim of the study was to investigate the relative importance of specific sociocultural factors, contextual factors, and psychological dimensions that were identified as being associated with resilience [23]. The second aim was to consider, for the first time, the potential impact on resilience of self-esteem, self-efficacy, and optimism in caregivers of people with dementia.

## Methods

### Design

The present study utilized baseline data from IDEAL, a longitudinal cohort study of people with dementia and caregivers. Details of the aims and procedures can be found in the programme protocol [15]. IDEAL was approved by the Wales Research Ethics Committee 5 (reference 13/WA/0405), the Scotland A Research Ethics Committee (reference 14/SS/0010) and the Ethics Committee of the School of Psychology, Bangor University (reference 2014–11684). IDEAL is registered with UK Clinical Research Network, registration number 16593. The present study utilized Version 6 of the baseline datasets. All participants gave written informed consent.

### Sample

Caregivers of people with dementia took part in IDEAL if the person with dementia they cared for also took part. People with dementia were recruited according to the inclusion criteria mentioned below and could nominate a caregiver to participate alongside them. Participation of a caregiver was not mandatory, and caregivers were free to choose whether to take part. A caregiver was defined as the primary person who provides practical or emotional unpaid support, usually a family member [8]. People with dementia were recruited through 29 National Health Service memory services and other specialist clinics, and via the online Join Dementia Research portal, between July 2014 and August 2016. At baseline the IDEAL cohort comprised 1537 people with dementia and 1277 caregivers. Inclusion criteria were a clinical diagnosis of any type of dementia, as judged by clinicians at recruitment sites, a Mini-Mental State Examination [25] score of 15 or above (indicating mild-to-moderate stages of dementia), and participants had to be residing in the community at the time of enrolment into the study [15]. There were no specific inclusion criteria for caregivers other than being available and willing to take part. An exclusion criterion for caregivers was the person with dementia withdrawing from the study at baseline before sufficient information was collected.

### Measures

In this study, resilience is defined as a composite index comprising the following five measures: a 10-item stressful life events schedule [26], the Neuropsychiatric Inventory-Questionnaire caregiver distress subscale [27], the Relative Stress Scale [28], the Role Captivity scale [29], and the Positive Aspects of Caregiving scale [30]. These measures extract from the IDEAL database indices of loss, hardship, burden, and adversity previously highlighted as contributors to resilience [18, 19, 21, 23]. For all measures other than the Positive Aspects of Caregiving scale, higher scores indicate a less positive caregiving experience; therefore, total scores for the Positive Aspects of Caregiving scale were reversed to be comparable to the scaling of the other composite resilience score measures. To give equal weight to each of the five measures total scores were then converted to a percentage [31]; see Supplementary Information 1 and Supplementary Tables 1 to 3 for a complete description of the measures and more detail concerning the construction of the composite resilience score. Each percentage score was combined, and a total percentage composite resilience score was computed. Scores for the composite resilience score therefore ranged between 0 and 100 with scores closer to zero indicating greater resilience.

The remaining measures included in the analysis were measures that broadly reflected either sociocultural, contextual, or psychological domains that were identified as moderating factors in three recent reviews [18, 19, 23]. These measures were self-rated by the caregiver except where noted. See Supplementary Information 2 for full details of these measures.

Sociocultural factors comprised sex, age, education, religiosity, ethnicity, and socio-economic status [32].

Contextual factors comprised caregiver status (spouse/partner vs. family/friend), dementia diagnostic type, informant-rated functional ability of the person with dementia [33–35], number of informant-rated neuropsychiatric symptoms of the person with dementia [27], social network [36], hours spent caregiving per day, the relationship quality of the caregiving dyad [37], social restriction [38], cultural capital [39], and time since dementia diagnosis. The latter was used as a proxy measure of how many years caregivers had been in their caregiving role.

Psychological dimensions comprised personality [40, 41], competence in the caregiving role [42], and management of meaning [29]. In addition to those factors, the present study also included measures of self-efficacy [43], optimism [44], and self-esteem [45].

### Planned analysis

To investigate whether resilience was associated with contextual factors and psychological dimensions, univariable and multiple regressions were employed. Variables were selected for inclusion in multiple regressions based on statistical significance after controlling for multiple comparisons and the size of the coefficient and 95% confidence intervals in univariable regressions. Multiple regressions were employed to investigate the combined contribution of important variables. For categorical variables the group with the largest sample size was used as the reference. Holm-Bonferroni correction for multiple comparisons was applied to all analyses.

Multiple imputation was conducted to account for missing data. For the composite measure multiple imputation was conducted at the item level. Ordinal variables were imputed using ordinal regression and categorical variables were imputed using multinomial regression. The imputed model included all variables in the analysis. Estimates from 25 imputed datasets were combined using Rubin’s rules [46].

## Results

Fifty-five caregivers did not provide any responses in at least one of the five composite resilience measures and therefore the sample used in the analysis comprised 1222 caregivers. Most caregivers were spouses/partners of the care recipients (81.9%), female (69.7%), and white British (96.1%), provided care for people with Alzheimer’s disease (55.8%), and provided at least one hour of care per day (77.5%), see Table 1. At the time of data collection, half of the caregivers had been caregiving for less than a year (50.8%), while a smaller percentage (1.6%) had been caregiving for six or more years. See Table 2 for mean scores on variables included in the analysis. The composite resilience score suggests that caregivers are generally resilient; see Supplementary Information 1 Fig. 1 for a distribution of frequency scores and Supplementary Information 1 Tables 2 and 3 for correlations and distribution of scores for each constituent percentage score.

**Table 1:** Characteristics of the sample

	Whole sample n = 1222
	N (%)
Caregiver sex	
Male	370 (30.3)
Female	852 (69.7)
Caregiver age	
<65	352 (28.8)
65–69	203 (16.6)
70–74	254 (20.8)
75–79	214 (17.5)
80+	199 (16.3)
Age; mean (SD)	69.02 (10.98)
Education	
No qualifications	260 (21.3)
School leaving certificate at age 16	275 (22.6)
School leaving certificate at age 18	365 (29.9)
University	318 (26.0)
Missing	4 (0.3)
Caregiver ethnicity	
White British	1174 (96.1)
White other	32 (2.6)
Other	11 (0.9)
Missing	5 (0.4)
Caregiver socio-economic status	
Higher managerial, administrative, & professional occupations	501 (41.0)
Intermediate occupations	422 (34.5)
Routine and manual occupations	256 (20.9)
Never worked and long-term unemployed	29 (2.4)
Missing	14 (1.1)
Caregiver status	
Spouse/partner	1001 (81.9)
Family/friend	221 (18.1)
Caregiver hours of caregiving (per day)	
<1 h	260 (21.3)
1–10 h	480 (39.3)
10 + hours	467 (38.2)
Missing	15 (1.2)
Person with dementia sex	
Male	726 (59.4)
Female	496 (40.6)
Person with dementia age	
<65	99 (8.1)
65–69	155 (12.7)
70–74	221 (18.1)
75–79	292 (23.9)
80+	455 (37.2)
Age; mean (SD)	76.12 (8.25)
Person with dementia diagnosis	
Alzheimer’s disease	682 (55.8)
Vascular dementia	134 (11.0)
Mixed Alzheimer’s and vascular	248 (20.3)
Frontotemporal dementia	44 (3.6)
Parkinson’s disease dementia	41 (3.4)
Dementia with Lewy bodies	42 (3.4)
Unspecified dementia/Other	31 (2.5)
Person with dementia time since diagnosis	
<1 year	621 (50.8)
1–2 years	367 (30.0)
3–5 years	127 (10.4)
6 + years	19 (1.6)
Missing	88 (7.2)

**Table 2:** Mean scores for variables of interest

	Mean (SD); n
Sociocultural factors	
Caregiver age	69.02 (10.98); 1222
Person with dementia age	76.12 (8.25); 1222
Importance of religiosity	3.03 (2.04); 1213
Contextual factors	
Functional ability	17.89 (8.57); 1154
Number of neuropsychiatric symptoms	3.60 (2.48); 1177
Social network	17.58 (5.54); 1193
Current relationship quality	23.11 (4.70); 1209
Social restriction	3.50 (1.35); 1211
Cultural capital	25.16 (5.48); 1158
Psychological dimensions	
Personality-Agreeableness	16.29 (2.61); 1215
Personality-Conscientiousness	15.52 (2.66); 1215
Personality-Extraversion	12.08 (3.34); 1211
Personality-Openness	13.21 (2.95); 1200
Personality-Neuroticism	10.88 (3.15); 1215
Caregiver competence	9.14 (1.67); 1214
Management of meaning	25.87 (4.40); 1191
Self-efficacy	31.63 (4.28); 1197
Optimism	14.69 (3.69); 1205
Self-esteem	31.14 (4.50); 1186
Resilience	
Stressful life events schedule	47.57 (47.44); 1222
Caregiver distress from neuropsychiatric symptoms	6.29 (6.42); 1033
Role captivity	5.55 (2.26); 1209
Relative Stress Scale	19.19 (9.83); 1180
Positive Aspects of Caregiving*	28.23 (7.40); 1216
Resilience composite percentage score	28.56 (10.11); 1000

* scores not reversed. Reversed mean scores (mean 25.78, SD 7.40)

**Table 3:** Univariable regressions and multiple regressions to identify factors associated with resilience in the whole sample: unstandardised regression coefficients and 95% confidence intervals

	Univariable regression	Multiple regression+
		Adj R^2^ = 0.564, *p* < .001
Variable	B (95% CI), *p* value	B (95% CI), *p* value
Sociocultural factors		
Caregiver sex	4.63 (3.44, 5.82), ***p*** **< .001**	2.23 (0.80, 3.67), ***p*** **= .002**
Caregiver age	-0.08 (-0.13, -0.03), ***p*** **= .002**	0.09 (0.05, 0.14), ***p <*** **.001**
Caregiver education		
No qualifications	-1.93 (-3.50, -0.35), *p* = .017	-1.65 (-2.79, -0.50), *p =* .005
School leaving certificate at age 16	0.72 (-0.83, 2.27), *p* = .364	-0.40 (-1.47, 0.67), *p* = .467
School leaving certificate at age 18	ref	ref
University	1.80 (0.30, 3.29), *p* = .018	0.99 (-0.11, 2.09), *p* = .076
Person with dementia sex	-3.86 (-4.98, -2.74), ***p*** **< .001**	-0.13 (-1.41, 1.14), *p* = .840
Person with dementia age	-0.07 (-0.13, 0.00), *p* = .062	-0.12 (-0.17, -0.06), ***p*** **< .001**
Caregiver ethnicity		
White British	ref	
White other	1.78 (-1.73, 5.29), *p* = .320	
Other	3.00 (-2.93, 8.93), *p* = .322	
Importance of religiosity	-0.08 (-0.35, 0.20), *p* = .594	
Caregiver socio-economic status		
Higher managerial, administrative, & professional occupations	ref	ref
Intermediate occupations	-0.94 (-2.23, 0.35), *p* = .154	0.07 (-0.89, 1.02), *p* = .895
Routine and manual occupations	-2.80 (-4.29, -1.31), ***p*** **< .001**	-1.09 (-2.26, 0.08), *p* = .068
Never worked and long-term unemployed	-1.05 (-4.74, 2.63), *p* = .575	-1.34 (-3.97, 1.30), *p* = .320
Contextual factors		
Caregiver status	-0.59 (-2.05, 0.87), *p* = .428	
Person with dementia diagnosis		
Alzheimer’s disease	ref	ref
Vascular dementia	0.19 (-1.65, 2.03), *p* = .841	0.06 (-1.19, 1.30), *p* = .929
Mixed Alzheimer’s and vascular	-0.03 (-1.47, 1.42), *p* = .970	0.02 (-0.97, 1.00), *p* = .975
Frontotemporal dementia	3.29 (0.26, 6.32), *p* = .033	-1.15 (-3.25, 0.94), *p* = .280
Parkinson’s disease dementia	3.82 (0.69, 6.96), *p* = .017	1.78 (-0.32, 3.89), *p* = .097
Dementia with Lewy bodies	4.23 (1.13, 7.33), *p* = .007	0.80 (-1.30, 2.90), *p* = .456
Unspecified dementia/Other	1.79 (-1.80, 5.38), *p* = .328	-0.42 (-2.83, 2.00), *p* = .736
Functional ability	0.45 (0.39, 0.51), ***p*** **< .001**	0.17 (0.11, 0.23), ***p*** **< .001**
Number of neuropsychiatric symptoms	2.10 (1.91, 2.29), ***p*** **< .001**	0.91 (0.73, 1.09), ***p*** **< .001**
Social network	-0.24 (-0.34, -0.14), ***p*** **< .001**	0.04 (-0.04, 0.11), *p* = .357
h of care provided per day		
<1 h	-4.62 (-6.08, -3.16), ***p*** **< .001**	-0.42 (-1.52, 0.68), *p* = .456
1–10 h	ref	ref
10 + hours	1.98 (0.72, 3.21), ***p*** **= .002**	0.85 (-0.06, 1.76), *p* = .068
Current relationship quality	-1.15 (-1.25, -1.05), ***p*** **< .001**	-0.62 (-0.71, -0.52), ***p*** **< .001**
Social restriction	2.54 (2.15, 2.93), ***p*** **< .001**	1.08 (0.77, 1.39), ***p*** **< .001**
Cultural capital	0.10 (0.01, 0.19), *p* = .034	
Person with dementia time since diagnosis		
< 1 year	ref	
1–2 years	1.82 (0.54, 3.10), *p* = .005	
3–5 years	2.61 (0.77, 4.45), *p* = .005	
6 + years	3.74 (0.41, 7.06), *p* = .028	
Caregiver employment status	-0.14 (-1.54, 1.27), *p* = .848	
Psychological dimensions		
Personality-Agreeableness	0.33 (0.12, 0.54), ***p*** **= .002**	0.07 (-0.09, 0.23), *p* = .411
Personality-Conscientiousness	-0.26 (-0.47, -0.05), *p* = .014	
Personality-Extraversion	-0.14 (-0.31, 0.03), *p* = .112	
Personality-Openness	0.34 (0.15, 0.53), ***p*** **< .001**	0.22 (0.07, 0.36), *p* = .003
Personality-Neuroticism	1.22 (1.05, 1.38), ***p*** **< .001**	0.46 (0.31, 0.61), ***p*** **< .001**
Caregiver competence	-2.66 (-2.96, -2.36), ***p*** **< .001**	-0.97 (-1.24, -0.70), ***p*** **< .001**
Management of Meaning	0.03 (-0.10, 0.15), *p* = .696	
Self-efficacy	-0.49 (-0.62, -0.37), ***p*** **< .001**	0.04 (-0.07, 0.15), *p* = .505
Optimism	-0.67 (-0.82, -0.52), ***p*** **< .001**	-0.17 (-0.31, -0.03), *p* = .018
Self-esteem	-0.71 (-0.82, -0.59), ***p*** **< .001**	0.00 (-0.11, 0.12), *p* = .949

+ all analyses adjusted for person with dementia and caregiver age, person with dementia and caregiver sex, caregiver education, and person with dementia diagnosis

Bold indicates significant at the 5% level after Holm-Bonferroni correction

For sociocultural factors using univariable analysis, after controlling for multiple comparisons, greater resilience in caregivers was associated with being a male caregiver and/or caregiving for a female person with dementia, being older, and having a routine or manual occupation rather than a higher status occupation; see Table 3. For contextual factors, for caregivers, greater resilience was associated with having a larger social network, rating the dyadic relationship quality higher, and experiencing fewer social restrictions; for people with dementia, greater caregiver resilience was associated with receiving less than one hour of care per day from the caregiver and having fewer informant-rated functional difficulties and neuropsychiatric symptoms. Whether the caregiver was a spouse/partner or a family/friend was unrelated to resilience, though the study may have been underpowered to test this effectively. For psychological dimensions having lower trait agreeableness, trait openness, and neuroticism, greater perceived caregiver competence, and higher self-efficacy, optimism, and self-esteem, were associated with greater resilience in the caregiver.

Including individually significant factors into a multiple regression, the final model accounted for 56% of the variance in resilience scores. After controlling for multiple comparisons greater resilience was significantly associated with sociocultural factors of being older and/or being a male caregiver and/or caregiving for an older person with dementia. Greater resilience was significantly associated with contextual factors of caregivers having fewer social restrictions, people with dementia having fewer informant-rated functional difficulties and neuropsychiatric symptoms, and better current quality of the relationship between the caregiver and the person with dementia. Psychological domain factors that remained significantly associated with greater caregiver resilience were lower trait neuroticism and greater perceived caregiver competence. Self-efficacy, optimism, self-esteem, agreeableness, openness, social network, socio-economic status, sex of the person with dementia, and number of hours spent caregiving were no longer significantly associated with caregiver resilience.

## Discussion

This study investigated factors associated with resilience in caregivers of people with dementia [23], and expanded the psychological element of previous models by including self-esteem, self-efficacy, and optimism. Based on the resilience composite from the current dataset, being older, male, and caregiving for an older person with dementia were the most important sociocultural factors. The most important contextual factors associated with greater resilience were fewer informant-rated functional difficulties and neuropsychiatric symptoms in the people with dementia, as well as the caregiver having fewer social restrictions and a better caregiver-rated dyadic relationship quality. From the psychological dimensions only lower caregiver neuroticism and higher perceived caregiver competence were associated with greater resilience. The present study also considered the contribution of self-esteem, self-efficacy, and optimism to resilience in caregivers. These three factors were individually significantly related to resilience; however, none remained associated with resilience in the multivariable model after correcting for multiple comparisons, suggesting that caregiver competence and neuroticism levels may be more important for greater resilience. The multivariable model of resilience explained 56% of the variance, confirming the predictive value of the composite, and suggesting that many of the key factors associated with resilience were included.

Of the factors that were suggested as being associated with greater resilience, being older, male, and caregiving for an older person with dementia were also the most important sociocultural factors identified in a recent review [23]. The observation of greater resilience in male caregivers was consistent with an earlier study of similar size to the present study [47]; and although it did not find an association with age, this had been reported in another study [21]. These differences could be due to how studies conceptualize and measure resilience as the latter focused on caregiver burden and difficulties of people with dementia that can affect caregiver burden [21] whereas the former focused on psychological well-being [47].

The most important contextual factors associated with greater resilience were people with dementia having fewer informant-rated functional difficulties and neuropsychiatric symptoms, as well as the caregiver having fewer social restrictions and the caregiver rating the dyadic relationship quality more positively. It is likely that these contextual factors are interrelated, as feeling less socially restricted could be due to people with dementia having fewer neuropsychiatric symptoms and requiring less support with everyday activities [18, 48]. Greater resilience in caregivers has been found to be indicative of two factors, higher care demands of the person with dementia and lower subjective caregiver burden [21]. In the present study informant-rated functional ability was generally moderate, though considerably higher than the cut-off for impairment [49], and the number of neuropsychiatric symptoms was also moderate. Caregiver stress was generally low in IDEAL [49], as were scores for most of the constituent variables that formed the composite resilience score. Indeed, the caregivers in the baseline assessment of IDEAL reported low subjective stress and few of the participants with mild-to-moderate dementia had high care needs. Therefore, it is possible that the associations between contextual factors and the resilience composite score reflects less stress in the caregiving role due to people with dementia having fewer difficulties. Lower scores on the resilience composite score were also associated with having fewer social restrictions and a more positive dyadic relationship, which could also indicate less caregiver burden. Indeed, positive dyadic relationships support well-being in caregivers of people with dementia [50]. Investigating how this resilience composite score changes over time as the care demands of people with dementia increase would be valuable.

From the caregiver psychological dimensions only lower neuroticism and higher perceived caregiver competence were associated with greater resilience. Competence has been considered one of the main personal assets needed for resilience [17] and it is likely that greater caregiver competence is related to lower levels of neuroticism [51]; therefore, caregivers who feel that they are doing a good job at caregiving and/or caregivers with lower neuroticism are likely to be more resilient when caregiving for people with mild-to-moderate dementia [52]. The present study also considered self-esteem, self-efficacy, and optimism and found that none of these factors remained associated with resilience in the multivariable model after correcting for multiple comparisons, suggesting that caregiver competence and neuroticism levels may be more important for resilience among caregivers of people with mild-to-moderate dementia. Again, it would be valuable to consider whether these relationships remain stable over time or contribute differently in longitudinal analyses. It is possible that as care needs increase with dementia severity, placing greater demands on caregivers’ psychological resources, factors such as self-esteem, self-efficacy and optimism may play a more significant role in supporting resilience.

Findings suggest that many of the factors proposed as being important in recent reviews of caregiver resilience [18, 19, 23] were independently related to resilience, but when combined in a multivariable regression model few remained significantly associated with the resilience composite score. In addition, as the model explained 56% of the variance it is likely that many of the important factors associated with greater resilience in caregivers were included; though 44% of the variance was still unexplained which suggests that there may be many smaller factors associated with resilience or a few factors that exert a large effect on caregiver resilience. Future research should focus on delineating the additional unexplained factors associated with resilience.

It is likely that different ways of investigating resilience account for differences in findings between studies, particularly as the majority of earlier studies used a single measure of resilience [24]. The present study approached this issue by creating a composite score; however, there is a clear need for a standardized measure of resilience designed specifically for caregivers of people with dementia that encompasses different constituents of resilience; this would increase understanding of the level of resilience and potentially help delineate consistent factors that associate with resilience in caregivers across different populations and different phases of caregiving. The findings of the present study suggest that resilience may not be the correct word to describe the situation of caregivers of people with mild-to-moderate dementia, as few caregivers indicated high levels of stress or burden and few people with dementia had high care needs. A standardized measure of resilience should focus on both the positive and negative aspects of caregiving, and consider aspects that fractionate caregivers into those reporting high and low burden and aspects that fractionate people with dementia into having high and low care demands. This will better help identify caregivers with high subjective burden who are caregiving for people with dementia with high care needs as these caregivers are likely to be at risk of low resilience and may need targeted support.

The study has some limitations that need to be considered. Resilience was measured with a composite score based on five measures concerning both positive and negative aspects of caregiving or current life situation. A percentage score was calculated to give equal weight to each of the five measures; however, correlations between the composite score and individual constituent parts suggest that the overall resilience score was less related to stressful life events than to other measures. This may be due to the ten selected stressful life events that were included; a third of the sample reported experiencing none of these stressful life events in the year prior to assessment and 60% had only experienced either one or two of these stressful life events. The resilience composite measure therefore may be more influenced by caregiver stress and role captivity than stressful life events. Creating a percentage score for each constituent measure prior to calculating an overall percentage score was intended to give equal weight to the five measures and to mitigate the effect any one measure had on the resilience score. A further limitation is that the study focused on caregivers of people with mild-to-moderate dementia. It may be harder to identify resilience in caregivers of this group where the demands of caregiving are relatively lower than for caregivers of people with more advanced dementia whose needs are greater [47]. Investigating resilience in caregivers as dementia severity increases would demonstrate whether there is a concomitant change in resilience as dementia severity increases.

## Conclusion

The present study found that there are factors relating to the caregiver and to the person with dementia that affect how resilient a dementia caregiver may be. It is apparent that caregiver resilience is not solely due to the personal assets and resources of the caregiver but may also be affected by the level of dependence of the person with dementia. Findings suggest that caregivers with high levels of neuroticism and low subjective caregiver competence, and who are caregiving for people with dementia with more impaired functional ability and have more neuropsychiatric symptoms, could benefit from greater support or targeted interventions designed to foster resilience. The findings suggest that resilience in caregivers of people with mild-to-moderate dementia is nuanced and that resilience may not be the correct term to describe their situation. Future research is needed to delineate whether caregivers of people with mild-to-moderate dementia are generally more resilient or whether people with mild-to-moderate dementia have fewer difficulties thus requiring less need for caregivers to demonstrate resilience.

### Electronic supplementary material

Below is the link to the electronic supplementary material.

Supplementary Material 1

Supplementary Material 2

## Data Availability

IDEAL data were deposited with the UK data archive in April 2020. Details of how to access the data can be found here: http://reshare.ukdataservice.ac.uk/854293/.
