# Supplementary material for: Resilience in caregivers of people with mild-to-moderate dementia: findings from the IDEAL cohort
Source: BMC Geriatr. 2023 Dec 5;23:804. doi: 10.1186/s12877-023-04549-y (PMC10696882; doi:10.1186/s12877-023-04549-y)
Supplement: Supplementary file 1 — Supplementary Material 1 [file 12877_2023_4549_MOESM1_ESM.docx]

**Resilience in carers of people with mild-to-moderate dementia: findings from the IDEAL cohort**

Supplementary Information 1

Resilience constituent measures

10-item stressful life events schedule

The Social Readjustment Rating Scale [1], known more commonly as the Holmes and Rahe Stress Scale, is a list of 49 stressful life events that have been linked to increased chances of becoming ill. In IDEAL an abbreviated version was used that contained 10 stressful life events that were most likely to be relevant to older people. These ten items are grouped under three general subheadings: bereavement, marital difficulties, and change in circumstances; see Supplementary Table 1 for each item. For the resilience score, the original Holmes and Rahe weightings were used to give a weighted score for the seriousness of each life event. These weights are included in the table below. The maximum possible total score was 538.

Supplementary Table 1. The ten items that comprise the stressful life events schedule

| Item | Weighting | N (%)* |
| --- | --- | --- |
| Death of spouse or child | 100 | 13 (1.1) |
| Death of a close family member (e.g. parent or sibling) | 63 | 164 (13.4) |
| Death of a close friend | 37 | 270 (22.1) |
| Divorce | 73 | 8 (0.7) |
| Marital Separation | 65 | 9 (0.7) |
| Retirement | 45 | 60 (4.9) |
| Moved home | 20 | 76 (6.2) |
| Major change in financial state (e.g. a lot worse off or a lot better off) | 38 | 108 (8.9) |
| Major change in health or behaviour of family member | 44 | 538 (44.0) |
| Major personal injury or illness | 53 | 63 (5.2) |
| None of the above | 0 | 399 (33.1) |

* Percentages do not total to 100 as more than one life event could be selected.

Relative Stress Scale

The Relative Stress Scale [2] is a widely-used 15-item self-report measure designed to assess the degree of distress and social upset experienced by a relative as the result of caring for a person with physical and/or behavioural difficulties. Total scores range from 0-60, with higher scores indicating more severe stress. The total score was included in the resilience composite score.

Neuropsychiatric Inventory Questionnaire

The Neuropsychiatric Inventory Questionnaire [3] addresses 12 neuropsychiatric domains: sleep, apathy, delusion, depression, anxiety, euphoria, agitation, eating/appetite, hallucination, disinhibition, irritability, and aberrant motor behaviour. Wording of the items is adapted from the United States National Alzheimer’s Coordinating Center Uniform Data Set [4]. For each of the 12 symptoms there is a yes/no screening question. Further questions rate the symptom severity of the person with dementia if the symptom is present and any emotional distress to the carer from the symptom being present in the person with dementia. Symptom severity was not included in the present study. Distress experienced by the carer in relation to each symptom was categorised as 0 (Not distressing at all), 1 (Minimal), 2 (Mild), 3 (Moderate), 4 (Severe), 6 (Extreme or very severe). Carer distress was included in the resilience score; therefore, total possible scores ranged between 0-72.

Role Captivity

Role Captivity [5] is a 3-item measure designed to assess the extent carers of people with dementia feel trapped in their role. The total scores range from 0 to 12, with higher scores indicating more role captivity.

Positive Aspects of Caregiving

The Positive Aspects of Caregiving scale [6] is a nine-item questionnaire that investigates positive aspects of being a carer such as whether providing help makes the carer feel useful. Respondents rate each item on a 5-point scale: 1 (Disagree a lot) to 5 (Agree a lot). Total scores range between 9 and 45, with higher scores indicating increased positive aspects. To make the total score suitable to convert into a percentage, 9 was subtracted from each total score which led to a converted scoring range of 0-36. As a higher score for this measure indicates a more positive caring experience total scores were reversed to match the scoring direction of the other measures included in the resilience measure; therefore, in the percentage score a higher score indicates fewer positive aspects of caring.

Calculating a composite resilience measure

Each total score was converted to a percentage using a formula that has been described previously [7]. Individual total scores for each measure were divided by the maximum possible score for that measure and this score was multiplied by 100 to form the percentage score.

These are the formulae for each of the constituent scores and the overall composite score:

Stressful life events: total score/538*100

Relative Stress Scale: total score/60*100

Neuropsychiatric Inventory Questionnaire-Carer Distress: total score/72*100

Role Captivity: total score/12*100

Positive Aspects of Caregiving: total score/36*100

The total composite score was the sum of these five percentage scores divided by 500 and then multiplied by 100.

Mean percentage scores for each constituent measure are shown in Supplementary Table 2. Correlations between each percentage score and the total composite score were calculated to investigate the relationship between the constituent scores and the total score; see Supplementary Table 2.

Supplementary Table 2. Correlations between constituent resilience variables and the composite resilience score

| Constituent resilience variable | Percentage score  Mean (SD) | Resilience composite score |
| --- | --- | --- |
| Stressful life events | 8.91 (8.89) | .273 |
| Relative Stress Scale | 32.20 (16.39) | .819 |
| Positive Aspects of Caregiving | 46.59 (16.44) | .633 |
| Role captivity | 46.36 (18.82) | .818 |
| Carer distress from neuropsychiatric symptoms | 9.64 (8.92) | .609 |

The individual constituent variables that contributed to the composite resilient score were further investigated and split into three groups to indicate severity level for each variable; low (0-33), moderate (34-66), and high (67-100). See Supplementary Table 3 for more information. Percentage scores were split into thirds to gauge distribution of percentage scores; lower scores indicate greater resilience and higher scores indicate less resilience. Supplementary Figure 1 shows for each measure the number of participants whose scores fall into each of the three severity levels.

Supplementary Table 3. Number of participants in each of the three severity levels for each variable

| Constituent resilience variable | Low 0-33 | Moderate 34-66 | High 67-100 |
| --- | --- | --- | --- |
| Stressful life events | 1196 | 25 | 1 |
| Relative Stress Scale | 636 | 556 | 30 |
| Positive Aspects of Caregiving | 254 | 784 | 184 |
| Role captivity | 261 | 734 | 227 |
| Carer distress from neuropsychiatric symptoms | 1193 | 29 | 0 |
| Resilience composite score | 839 | 382 | 1 |

Supplementary Figure 1. Frequency distribution of composite resilience scores


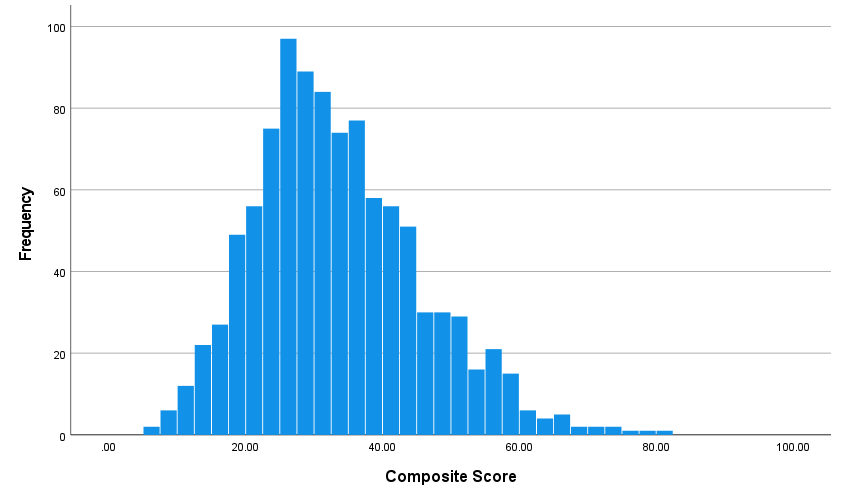


References

1. Holmes TH, Rahe RH: The Social Readjustment Rating Scale. *J Psychosom Res* 1967, 11(2):213-218. <https://doi.org/10.1016/0022-3999(67)90010-4>

2. Greene JG, Smith R, Gardiner M, Timbury GC: Measuring behavioural disturbance of elderly demented patients in the community and its effects on relatives: a factor analytic study. *Age Ageing* 1982, 11(2):121-126. <https://doi.org/10.1093/ageing/11.2.121>

3. Kaufer DI, Cummings JL, Ketchel P, Smith V, MacMillan A, Shelley T, Lopez OL, DeKosky ST: Validation of the NPI-Q, a brief clinical form of the Neuropsychiatric Inventory. *J Neuropsychiatry Clin Neurosci* 2000, 12(2):233-239. <https://doi.org/10.1176/appi.neuropsych.12.2.233>

4. Morris JC, National Alzheimer’s Coordinating Center: NACC Uniform Data Set (UDS) Coding Guidebook for Initial Visit Packet. Seattle: National Institute on Aging, ADC Clinical Task Force, NACC, University of Washington; 2008.

5. Pearlin LI, Mullan JT, Semple SJ, Skaff MM: Caregiving and the stress process: an overview of concepts and their measures. *Gerontologist* 1990, 30(5):583-594. <https://doi.org/10.1093/geront/30.5.583>

6. Tarlow BJ, Wisniewski SR, Belle SH, Rubert M, Ory MG, Gallagher-Thompson D: Positive Aspects of Caregiving contributions of the REACH project to the development of new measures for Alzheimer’s caregiving. *Res Aging* 2004, 26(4):429-453. <https://doi.org/10.1177/0164027504264493>

7. Martyr A, Clare L: Awareness of functional ability in people with early-stage dementia. *Int J Geriatr Psychiatry* 2018, 33(1):31-38. <https://doi.org/10.1002/gps.4664>
