# Supplementary material for: Resilience in caregivers of people with mild-to-moderate dementia: findings from the IDEAL cohort
Source: BMC Geriatr. 2023 Dec 5;23:804. doi: 10.1186/s12877-023-04549-y (PMC10696882; doi:10.1186/s12877-023-04549-y)
Supplement: Supplementary file 2 — Supplementary Material 2 [file 12877_2023_4549_MOESM2_ESM.docx]

**Resilience in carers of people with mild-to-moderate dementia: findings from the IDEAL cohort**

Supplementary Information 2

Other measures

Sociocultural factors

Age was grouped into five groups; <65, 65-69, 70-74, 75-79, 80+ for both people with dementia and caregivers. Mean age was included in regression analyses.

Sex was grouped into female and male.

Education was grouped into four groups; No qualifications, School leaving certificate at age 16, School leaving certificate at age 18, University.

Importance of religiosity was a single item that asked “How important is religion or being religious to your life?” Responses were: Not important, Slightly important, Somewhat important, Moderately important, Important, Very important, Extremely important. Total score ranges between 1 and 7. A higher score indicates that religiosity was more important.

Ethnicity was grouped into three groups; white British, white other, other. The sample were primarily White British which is representative of people with dementia and caregivers that attend memory clinics in Britain [1, 2].

Socio-economic status was grouped into four groups (Higher managerial, administrative, & professional occupations, Intermediate occupations, Routine and manual occupations,

Never worked and long-term unemployed) and was adapted from Office for National Statistics [3].

Contextual factors

Caregiver status was grouped into two groups; spouse/partner, family/friend.

Dementia diagnostic subtype was grouped into seven diagnostic groups; Alzheimer’s disease; vascular dementia; mixed-Alzheimer’s disease and vascular dementia; frontotemporal dementia; Parkinson’s disease dementia; dementia with Lewy bodies; unspecified/other.

Functional ability of the person with dementia was assessed using the 11-item amended informant-rated Functional Activities Questionnaire [4, 5]. The extra item was concerned with ability to use a telephone. This measure has been described in detail previously [6-8].

The number of neuropsychiatric symptoms present in the person with dementia was measured using the Neuropsychiatric Inventory [9, 10]. Total scores range between 0 and 12, with higher scores indicating the presence of more symptoms.

Social network was measured using the six-item Lubben Social Network Scale [11]. Total scores range between 0 and 30 with higher scores indicating larger social networks. Scores between 0 and 11 are indicative of being socially isolated.

The number of hours spent caring per day was grouped into three groups; <1 hour, 1-10 hours, 10+ hours.

Relationship quality was assessed using the five optional questions included in the Positive Affect Index [12]. These five questions address the current communication quality, closeness, similarity of views on life, engagement in joint activities, and overall relationship quality between the dyad. Total scores range between 5 and 30, with higher scores indicating better relationship quality.

Social restriction was assessed using the two-item Modified Social Restriction Scale [13]. This questionnaire asks how easy it is for the caregiver to find someone to look after the person with dementia if the caregiver was unwell, and if the caregiver needed a break from caregiving. Total scores range between 2 and 6, with higher total scores indicating greater social restriction.

Cultural capital was assessed using a 13-item scale that asks about frequency of engaging in a select group of 13 activities that include going to pubs, nightclubs, rock concerts, museums etc. [14]. This measure has been described in detail previously [15].

Time since dementia diagnosis was grouped into four groups; <1 year, 1-2 years, 3-5 years, 6+ years. This was based on the difference between the date of diagnosis and the date of the first assessment.

Psychological dimensions

Personality trait was assessed using the 20-item mini-IPIP [16]. This measure gives a score of each of the Big-Five personality traits; extraversion, agreeableness, conscientiousness, neuroticism, and intellect/imagination; the latter is referred to as openness in the manuscript. Total scores for each trait range between 5 and 20, with higher scores indicating higher levels of each personality trait [17].

Competence in the caregiving role was assed using the three-item Caregiving Competence Scale [18]. This measure was designed to assess the extent to which caregivers of people with dementia feel that they are doing an adequate job as a caregiver. Total scores range between 0 and 12, with higher scores indicating greater competence.

Management of Meaning is a nine-item measure that was designed to assess the extent caregivers of people with dementia feel that they have lost aspects of their personality because of caregiving [19]. Total scores range between 9 and 36, with higher scores indicating a more positive outlook on caregiving.

Self-efficacy was assessed using the 10-item Generalized Self-Efficacy Scale [20]. Total scores range between 10 and 40, with higher scores indicating higher levels of self-efficacy.

Optimism was assessed using the six non-filler items from the Life Orientation Test-Revised scale [21]. Total scores range between 0 and 24, with higher scores indicating more optimism.

Self-esteem was assessed using the 10-item Rosenberg Self-Esteem Scale [22]. The total score ranges from 10-40. A higher score is associated with greater self-esteem.

References

1. All-Party Parliamentary Group on Dementia: Dementia does not discriminate: the experiences of black, Asian and minority ethnic communities: House of Commons London; 2013.

2. Pham TM, Petersen I, Walters K, Raine R, Manthorpe J, Mukadam N, Cooper C: Trends in dementia diagnosis rates in UK ethnic groups: analysis of UK primary care data. *Clin Epidemiol* 2018, 10:949-960. <https://doi.org/10.2147/CLEP.S152647>

3. Office for National Statistics: Standard occupational classification 2010. Volume 3. The national statistics socioeconomic classification: (Rebased on the SOC2010) User Manual. Basingstoke: Palgrave Macmillan; 2010.

4. Martyr A, Clare L, Nelis SM, Marková IS, Roth I, Woods RT, Whitaker CJ, Morris RG: Verbal fluency and awareness of functional deficits in early-stage dementia. *Clin Neuropsychol* 2012, 26(3):501-519. <https://doi.org/10.1080/13854046.2012.665482>

5. Pfeffer RI, Kurosaki TT, Harrah CH, Jr., Chance JM, Filos S: Measurement of functional activities in older adults in the community. *J Gerontol* 1982, 37(3):323-329. <https://doi.org/10.1093/geronj/37.3.323>

6. Martyr A, Nelis SM, Clare L: Predictors of perceived functional ability in early-stage dementia: self-ratings, informant ratings and discrepancy scores. *Int J Geriatr Psychiatry* 2014, 29(8):852-862. <https://doi.org/10.1002/gps.4071>

7. Martyr A, Nelis SM, Quinn C, Rusted JM, Morris RG, Clare L, on behalf of the IDEAL programme team: The relationship between perceived functional difficulties and the ability to live well with mild-to-moderate dementia: findings from the IDEAL programme. *Int J Geriatr Psychiatry* 2019, 34(8):1251-1261. <https://doi.org/10.1002/gps.5128>

8. Martyr A, Gamble LD, Nelis SM, Collins R, Alexander CM, Morris RG, Quinn C, Pentecost C, Rusted JM, Victor C *et al*: Predictors of awareness of functional ability in people with dementia: the contribution of personality, cognition, and neuropsychiatric symptoms. Findings from the IDEAL programme. *Dement Geriatr Cogn Disord* 2022, 51(3):221-232. <https://doi.org/10.1159/000524607>

9. Morris JC, National Alzheimer’s Coordinating Center: NACC Uniform Data Set (UDS) Coding Guidebook for Initial Visit Packet. Seattle: National Institute on Aging, ADC Clinical Task Force, NACC, University of Washington; 2008.

10. Kaufer DI, Cummings JL, Ketchel P, Smith V, MacMillan A, Shelley T, Lopez OL, DeKosky ST: Validation of the NPI-Q, a brief clinical form of the Neuropsychiatric Inventory. *J Neuropsychiatry Clin Neurosci* 2000, 12(2):233-239. <https://doi.org/10.1176/appi.neuropsych.12.2.233>

11. Lubben J, Blozik E, Gillmann G, Iliffe S, von Renteln Kruse W, Beck JC, Stuck AE: Performance of an abbreviated version of the Lubben Social Network Scale among three European community-dwelling older adult populations. *Gerontologist* 2006, 46(4):503-513. <https://doi.org/10.1093/geront/46.4.503>

12. Bengtson VL, Schrader SS: Parent-child relations. In: *Research instruments in social gerontology: Social roles and social participation.* Edited by Mangon DJ, Peterson WA, vol. 2. Minnesota: University of Minnesota Press; 1982: 115-185.

13. Balducci C, Mnich E, McKee KJ, Lamura G, Beckmann A, Krevers B, Wojszel ZB, Nolan M, Prouskas C, Bień B *et al*: Negative impact and positive value in caregiving: validation of the COPE index in a six-country sample of carers. *Gerontologist* 2008, 48(3):276-286. <https://doi.org/10.1093/geront/48.3.276>

14. Thomson K: Cultural capital and social exclusion survey: technical report. London: National Centre for Social Research; 2004.

15. Sabatini S, Martyr A, Gamble LD, Jones IR, Collins R, Matthews FE, Victor C, Quinn C, Pentecost C, Thom JM *et al*: Profiles of social, cultural, and economic capital as longitudinal predictors of stress, positive experiences of caring, and depression among carers of people with dementia. *Aging Ment Health* 2022. <https://doi.org/10.1080/13607863.2022.2098920>

16. Donnellan MB, Oswald FL, Baird BM, Lucas RE: The mini-IPIP scales: tiny-yet-effective measures of the Big Five factors of personality. *Psychol Assess* 2006, 18(2):192-203. <https://doi.org/10.1037/1040-3590.18.2.192>

17. Hunt A, Martyr A, Gamble LD, Morris RG, Thom JM, Pentecost C, Clare L: The associations between personality traits and quality of life, satisfaction with life, and well-being over time in people with dementia and their caregivers: findings from the IDEAL programme. *BMC Geriatr* 2023, 23:354. <https://doi.org/10.1186/s12877-023-04075-x>

18. Robertson M, Zarit SH, Duncan LG, Rovine MJ, Femia EE: Family caregivers’ patterns of positive and negative affect. *Fam Relat* 2007, 56(1):12-23. <https://doi.org/10.1111/j.1741-3729.2007.00436.x>

19. Pearlin LI, Mullan JT, Semple SJ, Skaff MM: Caregiving and the stress process: an overview of concepts and their measures. *Gerontologist* 1990, 30(5):583-594. <https://doi.org/10.1093/geront/30.5.583>

20. Schwarzer R, Jerusalem M: Generalized Self-Efficacy Scale. In: *Measures in health psychology: a user’s portfolio Causal and control beliefs.* Edited by Weinman J, Wright S, Johnston M. Windsor, UK: NFER-NELSON; 1995: 35-37.

21. Scheier MF, Carver CS, Bridges MW: Distinguishing optimism from neuroticism (and trait anxiety, self-mastery, and self-esteem): a reevaluation of the Life Orientation Test. *J Pers Soc Psychol* 1994, 67(6):1063-1078. <https://doi.org/10.1037/0022-3514.67.6.1063>

22. Rosenberg M: Society and the adolescent self-image. Princeton, NJ: Princeton University Press; 1965.
